# Poly[[aqua­{μ_4_-2-[(carb­oxy­meth­yl)sulfan­yl]nicotinato-κ^4^ *O*:*O*′:*O*′′:*O*′′′}copper(II)] trihydrate]

**DOI:** 10.1107/S1600536813009604

**Published:** 2013-04-13

**Authors:** Wei-Qi Li

**Affiliations:** a Jinhua Radio and Television University, Zhejiang 321022, People’s Republic of China

## Abstract

In the polymeric title complex, {[Cu(C_8_H_5_NO_4_S)(H_2_O)]·3H_2_O}_*n*_, the Cu^II^ cation is coordinated by one water mol­ecule and four carboxyl­ate O atoms from four 2-[(carb­oxy­meth­yl)sulfan­yl]nicotinate anions in a distorted square-pyramidal geometry. The 2-[(carb­oxy­meth­yl)sulfan­yl]nicotinate anion bridges four Cu^II^ cations, forming a two-dimensional polymeric complex parallel to the *bc* plane. In the crystal, O—H⋯O, O—H⋯N and O—H⋯S hydrogen bonds link the complex mol­ecules and lattice water mol­ecules into a three-dimensional supra­molecular architecture.

## Related literature

For background to the 2-[(carb­oxy­meth­yl)sulfan­yl]nicotinato ligand, see: Wang & Feng (2010 ▶). For related compounds, see: Jiang *et al.* (2012 ▶). For metal complexes with 2-mercaptonanicotinate ligands, see: Humphrey *et al.* (2006 ▶); Sun *et al.* (2011 ▶).
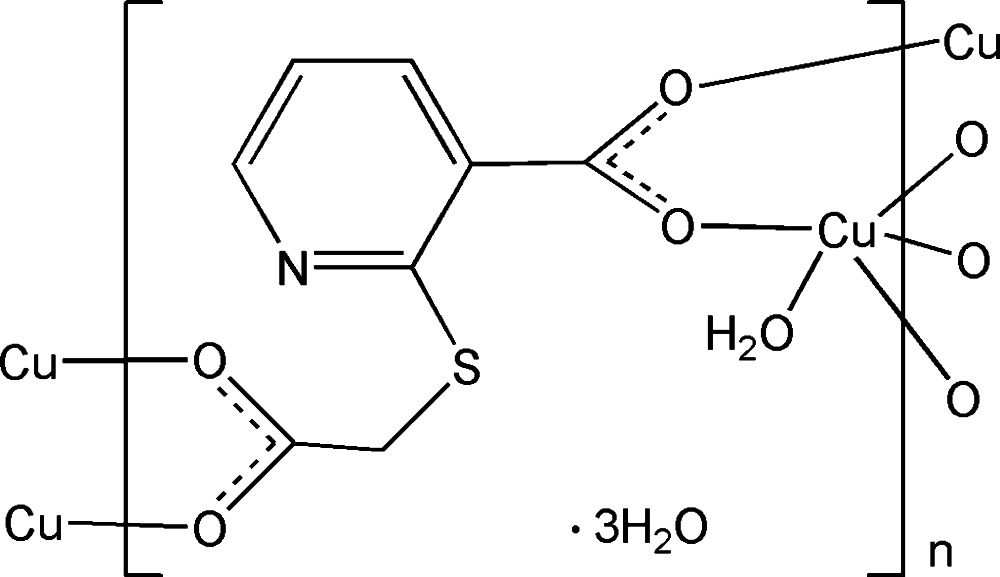


## Experimental

### 

#### Crystal data

[Cu(C_8_H_5_NO_4_S)(H_2_O)]·3H_2_O
*M* *_r_* = 346.82
Monoclinic, 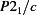

*a* = 9.940 (7) Å
*b* = 16.639 (9) Å
*c* = 7.876 (4) Å
β = 96.28 (5)°
*V* = 1294.8 (13) Å^3^
*Z* = 4
Mo *K*α radiation
μ = 1.88 mm^−1^
*T* = 296 K
0.25 × 0.09 × 0.06 mm

#### Data collection

Bruker SMART APEXII area-detector diffractometer
Absorption correction: multi-scan (*SADABS*; Bruker, 2001 ▶) *T*_min_ = 0.812, *T*_max_ = 0.892
20324 measured reflections
2973 independent reflections
2331 reflections with *I* > 2σ(*I*)
*R*_int_ = 0.054

#### Refinement

*R*[*F* ^2^ > 2σ(*F* ^2^)] = 0.032
*wR*(*F* ^2^) = 0.085
*S* = 1.04
2973 reflections
172 parameters
H-atom parameters constrained
Δρ_max_ = 0.37 e Å^−3^
Δρ_min_ = −0.32 e Å^−3^

### 

Data collection: *APEX2* (Bruker, 2007 ▶); cell refinement: *SAINT* (Bruker, 2007 ▶); data reduction: *SAINT*; program(s) used to solve structure: *SHELXS97* (Sheldrick, 2008 ▶); program(s) used to refine structure: *SHELXL97* (Sheldrick, 2008 ▶); molecular graphics: *SHELXTL* (Sheldrick, 2008 ▶); software used to prepare material for publication: *SHELXL97*.

## Supplementary Material

Crystal structure: contains datablock(s) I, global. DOI: 10.1107/S1600536813009604/xu5692sup1.cif

Structure factors: contains datablock(s) I. DOI: 10.1107/S1600536813009604/xu5692Isup2.hkl

Supplementary material file. DOI: 10.1107/S1600536813009604/xu5692Isup3.cdx

Additional supplementary materials:  crystallographic information; 3D view; checkCIF report

## Figures and Tables

**Table 1:** Selected bond lengths (Å)

Cu1—O1	1.958 (2)
Cu1—O2^i^	1.9682 (19)
Cu1—O3^ii^	1.9687 (19)
Cu1—O4^iii^	1.9836 (19)
Cu1—O1*W*	2.171 (2)

Symmetry codes: (i) 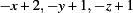; (ii) 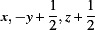; (iii) 
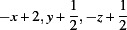
.

**Table 2:** Hydrogen-bond geometry (Å, °)

*D*—H⋯*A*	*D*—H	H⋯*A*	*D*⋯*A*	*D*—H⋯*A*
O1*W*—H1*WA*⋯O4*W* ^iv^	0.82	1.93	2.747 (4)	174
O1*W*—H1*WB*⋯O2*W* ^iii^	0.84	2.11	2.899 (4)	156
O2*W*—H2*WA*⋯O3*W* ^v^	0.83	2.05	2.843 (5)	157
O2*W*—H2*WB*⋯O4	0.84	2.26	2.911 (4)	135
O2*W*—H2*WB*⋯N1	0.84	2.56	3.253 (4)	141
O3*W*—H3*WA*⋯O3^vi^	0.82	2.40	3.110 (4)	146
O3*W*—H3*WB*⋯O2*W*	0.83	2.00	2.803 (5)	165
O4*W*—H4*WB*⋯S1^vi^	0.85	2.62	3.356 (4)	146

Symmetry codes: (iii) 
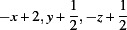
; (iv) 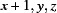; (v) 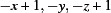; (vi) 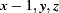.

**Fig. 1.**
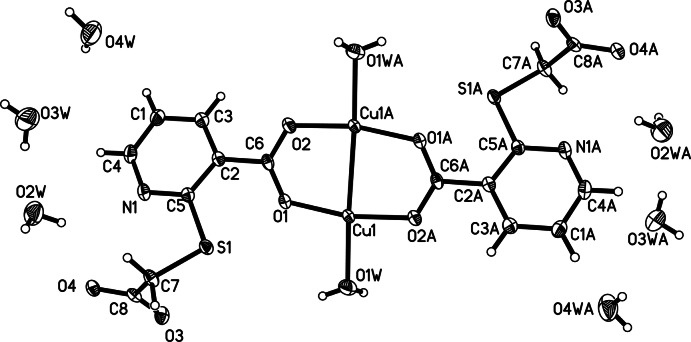
A view of the molecule of (I) showing the atom-labelling scheme with displacement ellipsoids drawn at the 30% probability. Symmetry codes: (A) -x + 2,-y + 1,-z + 1.

**Fig. 2.**
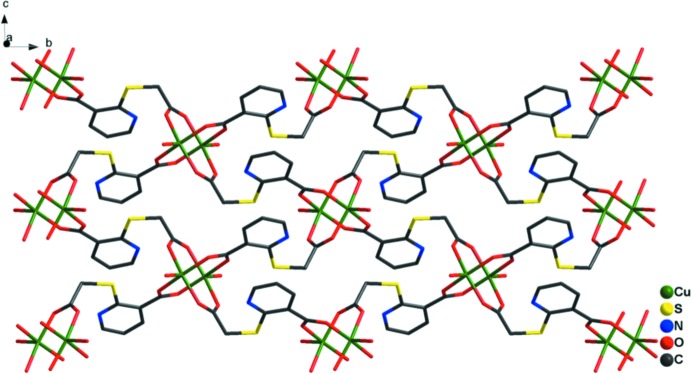
A view of the two-dimensional layer structure of (I).

**Fig. 3.**
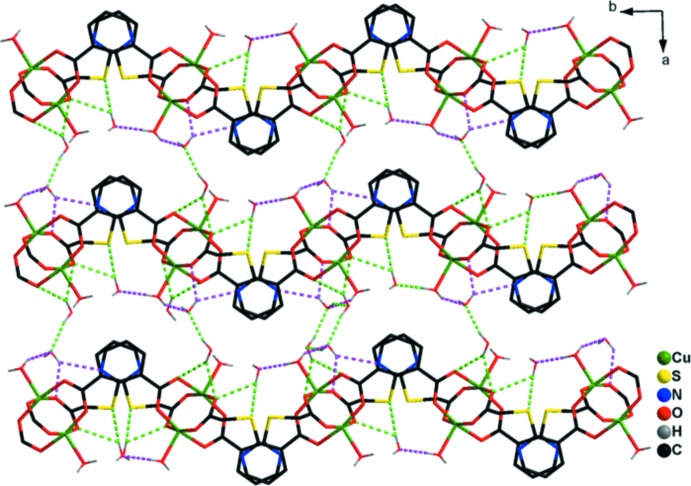
three-dimensional supramolecular architecture of (I). Dashed lines indicate hydrogen bonds.

## supplementary crystallographic information

### Comment

2-Carboxymethylsulfanylnicotinic acid is prepared from 2-mercaptonanicotinic acid (Wang & Feng, 2010). 2-Mercaptonanicotinic acid is a multifunctional ligand, and some complexes containing 2-mercaptonanicotinate ligand have been previously investigated (Humphrey *et al.*, 2006; Sun *et al.*, 2011).

The 2-carboxymethylsulfanylnicotinic acid is an interesting ligand because of its potential versatile coordinate behavior. Recently, only three metal compounds have been reported about 2-carboxymethylsulfanyl nicotinic acid (Jiang *et al.*, 2012). Herein, we report the synthesis and structure of the title compound.

A perspective view of (I) is presented in Fig.1. The asymmetric unit consists of one Cu^II^ ion, one (C_8_H_5_NO_4_S)^2-^ ligands, one coordinated water molecule, and three lattice water molecules. As shown in Fig. 2, each pair of Cu^2+^ ion is *µ*-linked by four carboxylic groups of the individual (C_8_H_5_NO_4_S)^2-^ ligands with Cu···Cu distances of 2.6524 (15) Å. The (C_8_H_5_NO_4_S)^2-^ ligands bridge adjacent dinuclear units in a head-to-tail fashion to form a two-dimensional layer on the *bc* plane, and further linked into the three-dimensional architecture by O—H···O/N/S hydrogen bonds (Fig.3).

### Experimental

2-Carboxymethylsulfanyl nicotinic acid (1.0 mmol) in H_2_O (10 ml) was stirred under basic condition in which NH_3_.H_2_O was needed to keep pH value of 11. CuCl_2_.2H_2_O was added and stirred for 2 h. The resulting solution was placed for 2 days, and the crystals were filtered off, giving blue crystals of the title compound for X-ray analysis.

### Refinement

The carbon-bound H-atoms were positioned geometrically and included in the refinement using a riding model [aromatic C—H 0.93 Å and aliphatic C—H 0.97 Å, *U*_iso_(H) = 1.2*U*_eq_(C)]. The oxygen-bound H-atoms was located in a difference Fourier maps and refined with the O—H distance restrained to 0.83 (2) Å and *U*_iso_(H) = 1.2*U*_eq_(O).

### Crystal data

**Table:**

[Cu(C_8_H_5_NO_4_S)(H_2_O)]·3H_2_O	*F*(000) = 708
*M**_r_* = 346.82	*D*_x_ = 1.779 Mg m^−^^3^
Monoclinic, *P*2_1_/*c*	Mo *K*α radiation, λ = 0.71073 Å
Hall symbol: -P 2ybc	Cell parameters from 3501 reflections
*a* = 9.940 (7) Å	θ = 2.1–27.6°
*b* = 16.639 (9) Å	µ = 1.88 mm^−^^1^
*c* = 7.876 (4) Å	*T* = 296 K
β = 96.28 (5)°	Plate, blue
*V* = 1294.8 (13) Å^3^	0.25 × 0.09 × 0.06 mm
*Z* = 4	

### Data collection

**Table:**

Bruker SMART APEXII area-detector diffractometer	2973 independent reflections
Radiation source: fine-focus sealed tube	2331 reflections with *I* > 2σ(*I*)
Graphite monochromator	*R*_int_ = 0.054
ω scans	θ_max_ = 27.6°, θ_min_ = 2.1°
Absorption correction: multi-scan (*SADABS*; Bruker, 2001)	*h* = −11→12
*T*_min_ = 0.812, *T*_max_ = 0.892	*k* = −21→21
20324 measured reflections	*l* = −10→10

### Refinement

**Table:**

Refinement on *F*^2^	Primary atom site location: structure-invariant direct methods
Least-squares matrix: full	Secondary atom site location: difference Fourier map
*R*[*F*^2^ > 2σ(*F*^2^)] = 0.032	Hydrogen site location: inferred from neighbouring sites
*wR*(*F*^2^) = 0.085	H-atom parameters constrained
*S* = 1.04	*w* = 1/[σ^2^(*F*_o_^2^) + (0.0455*P*)^2^ + 0.2317*P*] where *P* = (*F*_o_^2^ + 2*F*_c_^2^)/3
2973 reflections	(Δ/σ)_max_ = 0.001
172 parameters	Δρ_max_ = 0.37 e Å^−^^3^
0 restraints	Δρ_min_ = −0.32 e Å^−^^3^

### Special details

**Table:**

Geometry. All e.s.d.'s (except the e.s.d. in the dihedral angle between two l.s. planes) are estimated using the full covariance matrix. The cell e.s.d.'s are taken into account individually in the estimation of e.s.d.'s in distances, angles and torsion angles; correlations between e.s.d.'s in cell parameters are only used when they are defined by crystal symmetry. An approximate (isotropic) treatment of cell e.s.d.'s is used for estimating e.s.d.'s involving l.s. planes.
Refinement. Refinement of *F*^2^ against ALL reflections. The weighted *R*-factor *wR* and goodness of fit *S* are based on *F*^2^, conventional *R*-factors *R* are based on *F*, with *F* set to zero for negative *F*^2^. The threshold expression of *F*^2^ > σ(*F*^2^) is used only for calculating *R*-factors(gt) *etc*. and is not relevant to the choice of reflections for refinement. *R*-factors based on *F*^2^ are statistically about twice as large as those based on *F*, and *R*- factors based on ALL data will be even larger.

### Fractional atomic coordinates and isotropic or equivalent isotropic displacement parameters (Å^2^)

**Table:**

	*x*	*y*	*z*	*U*_iso_*/*U*_eq_	
Cu1	1.10928 (3)	0.455426 (15)	0.48878 (4)	0.02469 (11)	
S1	0.96122 (7)	0.21713 (3)	0.44052 (8)	0.03032 (16)	
O1	0.97500 (18)	0.37850 (10)	0.3866 (2)	0.0338 (4)	
O1W	1.2932 (2)	0.39085 (12)	0.4498 (3)	0.0473 (5)	
H1WA	1.2787	0.3469	0.4037	0.057*	
H1WB	1.3419	0.4141	0.3845	0.057*	
O2	0.7893 (2)	0.45444 (10)	0.3972 (3)	0.0369 (4)	
O3	1.0779 (2)	0.08898 (11)	0.2128 (2)	0.0374 (4)	
O4	0.89346 (19)	0.01319 (10)	0.2319 (2)	0.0328 (4)	
N1	0.7301 (2)	0.17934 (12)	0.2526 (3)	0.0341 (5)	
C1	0.5606 (3)	0.27286 (16)	0.1366 (4)	0.0398 (7)	
H1A	0.4768	0.2817	0.0745	0.048*	
C2	0.7636 (3)	0.32137 (14)	0.2965 (3)	0.0267 (5)	
C3	0.6384 (3)	0.33602 (16)	0.2060 (4)	0.0348 (6)	
H3A	0.6068	0.3885	0.1919	0.042*	
C4	0.6114 (3)	0.19624 (16)	0.1627 (4)	0.0393 (7)	
H4A	0.5600	0.1536	0.1147	0.047*	
C5	0.8059 (3)	0.24081 (14)	0.3179 (3)	0.0260 (5)	
C6	0.8489 (3)	0.38985 (13)	0.3664 (3)	0.0268 (5)	
C7	0.9514 (3)	0.10887 (14)	0.4500 (3)	0.0319 (6)	
H7A	1.0178	0.0897	0.5403	0.038*	
H7B	0.8626	0.0938	0.4794	0.038*	
C8	0.9757 (3)	0.06765 (14)	0.2838 (3)	0.0286 (5)	
O2W	0.6123 (3)	−0.00071 (16)	0.2997 (4)	0.0759 (8)	
H2WA	0.6385	−0.0244	0.3907	0.091*	
H2WB	0.6774	0.0299	0.2886	0.091*	
O3W	0.3765 (3)	0.07734 (18)	0.3764 (4)	0.0831 (9)	
H3WA	0.3048	0.0589	0.3329	0.100*	
H3WB	0.4364	0.0491	0.3424	0.100*	
O4W	0.2538 (3)	0.24845 (18)	0.2749 (5)	0.1001 (11)	
H4WA	0.2766	0.2280	0.1881	0.120*	
H4WB	0.1699	0.2362	0.2691	0.120*	

### Atomic displacement parameters (Å^2^)

**Table:**

	*U*^11^	*U*^22^	*U*^33^	*U*^12^	*U*^13^	*U*^23^
Cu1	0.02945 (19)	0.01710 (15)	0.02766 (18)	0.00031 (11)	0.00377 (12)	0.00057 (11)
S1	0.0364 (4)	0.0231 (3)	0.0307 (4)	0.0012 (3)	0.0001 (3)	−0.0052 (2)
O1	0.0335 (11)	0.0208 (8)	0.0472 (12)	−0.0032 (7)	0.0043 (9)	−0.0036 (7)
O1W	0.0429 (12)	0.0403 (11)	0.0603 (14)	0.0085 (9)	0.0123 (10)	−0.0024 (9)
O2	0.0382 (11)	0.0238 (9)	0.0469 (12)	0.0011 (8)	−0.0026 (9)	−0.0080 (8)
O3	0.0434 (12)	0.0363 (10)	0.0337 (10)	−0.0079 (9)	0.0093 (9)	−0.0118 (8)
O4	0.0412 (11)	0.0248 (8)	0.0330 (10)	−0.0010 (8)	0.0066 (8)	−0.0051 (7)
N1	0.0417 (14)	0.0242 (10)	0.0357 (13)	−0.0065 (9)	0.0016 (10)	−0.0041 (9)
C1	0.0335 (16)	0.0393 (15)	0.0444 (17)	−0.0048 (12)	−0.0052 (13)	−0.0017 (12)
C2	0.0320 (14)	0.0234 (12)	0.0254 (13)	−0.0021 (10)	0.0056 (10)	−0.0014 (9)
C3	0.0357 (16)	0.0275 (13)	0.0410 (16)	−0.0008 (11)	0.0027 (12)	0.0011 (11)
C4	0.0424 (17)	0.0338 (14)	0.0403 (16)	−0.0121 (12)	−0.0012 (13)	−0.0045 (12)
C5	0.0308 (14)	0.0242 (11)	0.0238 (12)	−0.0016 (10)	0.0066 (10)	−0.0016 (9)
C6	0.0398 (16)	0.0188 (11)	0.0221 (12)	−0.0042 (10)	0.0039 (11)	0.0023 (9)
C7	0.0499 (17)	0.0225 (12)	0.0234 (13)	0.0016 (11)	0.0039 (12)	−0.0010 (9)
C8	0.0388 (15)	0.0202 (11)	0.0263 (13)	0.0065 (10)	0.0015 (11)	−0.0008 (9)
O2W	0.0679 (18)	0.0680 (17)	0.097 (2)	−0.0153 (14)	0.0337 (15)	−0.0023 (15)
O3W	0.0590 (18)	0.0867 (19)	0.098 (2)	−0.0033 (15)	−0.0178 (15)	−0.0172 (17)
O4W	0.069 (2)	0.090 (2)	0.150 (3)	−0.0265 (17)	0.048 (2)	−0.055 (2)

### Geometric parameters (Å, º)

**Table:**

Cu1—O1	1.958 (2)	N1—C5	1.340 (3)
Cu1—O2^i^	1.9682 (19)	C1—C4	1.379 (4)
Cu1—O3^ii^	1.9687 (19)	C1—C3	1.382 (4)
Cu1—O4^iii^	1.9836 (19)	C1—H1A	0.9300
Cu1—O1W	2.171 (2)	C2—C3	1.386 (4)
Cu1—Cu1^i^	2.6524 (15)	C2—C5	1.410 (3)
S1—C5	1.773 (3)	C2—C6	1.489 (3)
S1—C7	1.806 (3)	C3—H3A	0.9300
O1—C6	1.261 (3)	C4—H4A	0.9300
O1W—H1WA	0.8228	C7—C8	1.521 (3)
O1W—H1WB	0.8384	C7—H7A	0.9700
O2—C6	1.263 (3)	C7—H7B	0.9700
O2—Cu1^i^	1.9682 (19)	O2W—H2WA	0.8345
O3—C8	1.263 (3)	O2W—H2WB	0.8359
O3—Cu1^iv^	1.9687 (19)	O3W—H3WA	0.8165
O4—C8	1.258 (3)	O3W—H3WB	0.8253
O4—Cu1^v^	1.9836 (19)	O4W—H4WA	0.8176
N1—C4	1.337 (4)	O4W—H4WB	0.8547
			
O1—Cu1—O2^i^	167.93 (8)	C3—C2—C5	117.9 (2)
O1—Cu1—O3^ii^	87.44 (9)	C3—C2—C6	119.9 (2)
O2^i^—Cu1—O3^ii^	90.03 (9)	C5—C2—C6	122.2 (2)
O1—Cu1—O4^iii^	90.75 (9)	C1—C3—C2	120.1 (2)
O2^i^—Cu1—O4^iii^	89.31 (9)	C1—C3—H3A	119.9
O3^ii^—Cu1—O4^iii^	168.14 (8)	C2—C3—H3A	119.9
O1—Cu1—O1W	99.49 (9)	N1—C4—C1	124.1 (3)
O2^i^—Cu1—O1W	92.56 (9)	N1—C4—H4A	117.9
O3^ii^—Cu1—O1W	99.18 (9)	C1—C4—H4A	117.9
O4^iii^—Cu1—O1W	92.69 (9)	N1—C5—C2	122.1 (2)
O1—Cu1—Cu1^i^	82.33 (7)	N1—C5—S1	117.35 (19)
O2^i^—Cu1—Cu1^i^	85.74 (7)	C2—C5—S1	120.48 (19)
O3^ii^—Cu1—Cu1^i^	86.50 (7)	O1—C6—O2	125.7 (2)
O4^iii^—Cu1—Cu1^i^	81.64 (7)	O1—C6—C2	116.7 (2)
O1W—Cu1—Cu1^i^	174.09 (6)	O2—C6—C2	117.5 (2)
C5—S1—C7	101.32 (12)	C8—C7—S1	113.56 (17)
C6—O1—Cu1	125.29 (16)	C8—C7—H7A	108.9
Cu1—O1W—H1WA	113.2	S1—C7—H7A	108.9
Cu1—O1W—H1WB	114.3	C8—C7—H7B	108.9
H1WA—O1W—H1WB	103.0	S1—C7—H7B	108.9
C6—O2—Cu1^i^	120.56 (18)	H7A—C7—H7B	107.7
C8—O3—Cu1^iv^	120.36 (16)	O4—C8—O3	125.7 (2)
C8—O4—Cu1^v^	125.43 (17)	O4—C8—C7	116.5 (2)
C4—N1—C5	118.0 (2)	O3—C8—C7	117.8 (2)
C4—C1—C3	117.6 (3)	H2WA—O2W—H2WB	101.8
C4—C1—H1A	121.2	H3WA—O3W—H3WB	106.2
C3—C1—H1A	121.2	H4WA—O4W—H4WB	102.4

Symmetry codes: (i) −*x*+2, −*y*+1, −*z*+1; (ii) *x*, −*y*+1/2, *z*+1/2; (iii) −*x*+2, *y*+1/2, −*z*+1/2; (iv) *x*, −*y*+1/2, *z*−1/2; (v) −*x*+2, *y*−1/2, −*z*+1/2.

### Hydrogen-bond geometry (Å, º)

**Table:**

*D*—H···*A*	*D*—H	H···*A*	*D*···*A*	*D*—H···*A*
O1*W*—H1*WA*···O4*W*^vi^	0.82	1.93	2.747 (4)	174
O1*W*—H1*WB*···O2*W*^iii^	0.84	2.11	2.899 (4)	156
O2*W*—H2*WA*···O3*W*^vii^	0.83	2.05	2.843 (5)	157
O2*W*—H2*WB*···O4	0.84	2.26	2.911 (4)	135
O2*W*—H2*WB*···N1	0.84	2.56	3.253 (4)	141
O3*W*—H3*WA*···O3^viii^	0.82	2.40	3.110 (4)	146
O3*W*—H3*WB*···O2*W*	0.83	2.00	2.803 (5)	165
O4*W*—H4*WB*···S1^viii^	0.85	2.62	3.356 (4)	146

Symmetry codes: (iii) −*x*+2, *y*+1/2, −*z*+1/2; (vi) *x*+1, *y*, *z*; (vii) −*x*+1, −*y*, −*z*+1; (viii) *x*−1, *y*, *z*.
